# A rapid review of evidence relating to service use, experiences, and support needs of adults from minority ethnic communities along the eyecare pathway in the United Kingdom

**DOI:** 10.3389/fpubh.2023.1119540

**Published:** 2023-02-28

**Authors:** Nikki Heinze, Lee Jones, Bhavini Makwana

**Affiliations:** ^1^BRAVO VICTOR Research, London, United Kingdom; ^2^UCL Institute of Ophthalmology, London, United Kingdom; ^3^BAME Vision, London, United Kingdom

**Keywords:** visual impairment, sight loss, minority ethnic, social inequalities, health inequalities, BAME

## Abstract

**Background:**

There is growing awareness of the health inequalities experienced by minority ethnic communities, who make up an increasing proportion of the United Kingdom (UK) population and have been found to be at increased risk of visual impairment (V.I.). V.I. impacts on a wide range of life domains including employment, social functioning and activities of daily living. Considering existing health inequalities, the increased risk of V.I. and its wide-ranging impact, it is important to understand the experiences of adults from minority ethnic communities living with V.I. in the UK.

**Methods:**

A rapid evidence review of academic and gray literature published since 2005 and in English was performed. A search of AMED, CINAHL Plus and MEDLINE *via* EBSCOhost identified 969 articles. Articles were included in the review if they reported findings relating to the UK-context, to adults from minority ethnic communities living with V.I., and to experiences of V.I. and the eyecare pathway.

**Results:**

A total of 11 academic articles and 4 charity reports presented findings relating to perceptions of V.I. and eye disease (*n* = 3), access to services and service use (*n* = 5), impact of interventions (*n* = 7), the wider impact of V.I. (*n* = 2), and registration status (*n* = 1). Much of the literature focused on primary eyecare resulting in a comprehensive list of barriers and recommendations to increase eye tests. Less research addressed experiences and use of services further along the eyecare pathway although use of services may be low. Overall, the research on the experiences of adults with V.I. from minority ethnic communities in the UK remains anecdotal, outdated or unavailable. There are substantial gaps in the evidence relating to the wider impact of V.I., the impact of perceptions of V.I., and the use of services beyond primary eyecare.

**Conclusions:**

This review summarizes our current knowledge of the experiences of adults from minority ethnic communities living with V.I. in the UK and highlights substantial gaps in the evidence. The findings provide practical implications for practitioners and researchers committed to addressing health inequalities in the field of eyecare in the UK.

## 1. Introduction

**Figure 1:**
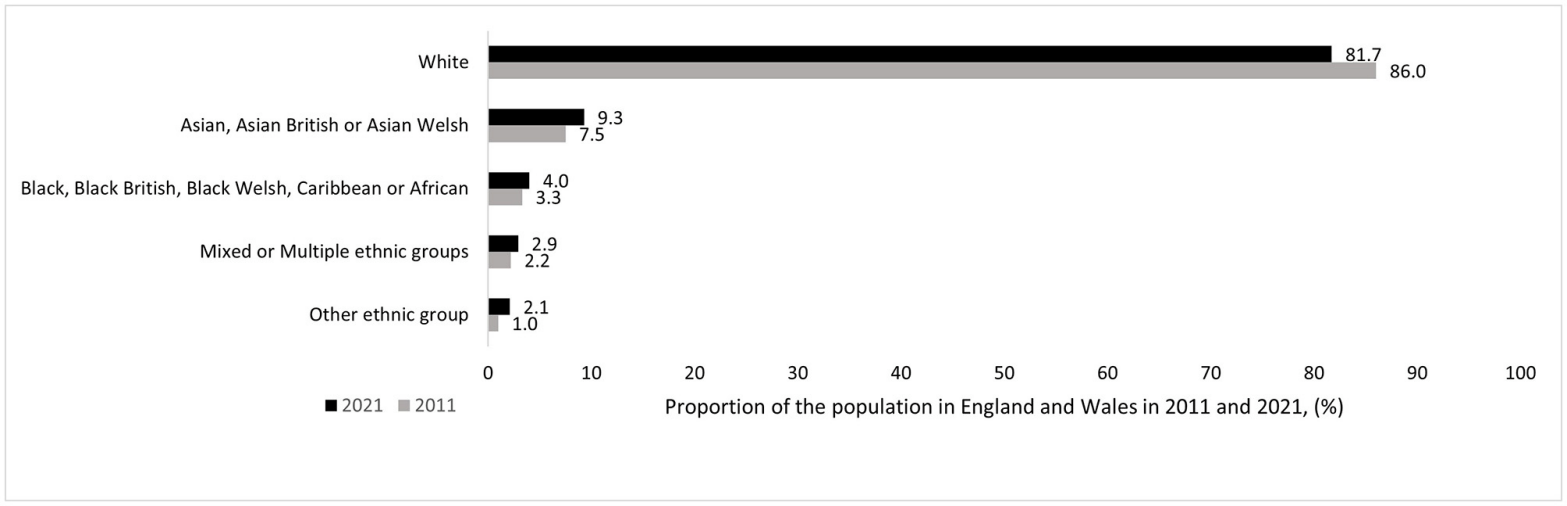
Change in ethnic make-up of usual residents in England and Wales between 2011 and 2021 (2).

In 2011, minority ethnic groups made up ~13% of the UK population (1). There is evidence that since then the proportion of people from all minority ethnic communities has increased in England and Wales, whilst the proportion of those identifying as white has decreased (Figure 1) (2). The UK Census provides the following ethnic groups: Asian (including Bangladeshi, Chinese, Indian, Pakistani and other Asian), black (including African, Caribbean, and other black), mixed or multiple ethnic groups (including white and Asian, white and black African, white and black Caribbean and other mixed or multiple ethnic groups), white (including English, Welsh, Scottish, Northern Irish or British, Irish, Gypsy or Irish Traveler, Roma and other white) and other ethnic group (including Arab and any other ethnic group) (2). However, in the context of research, small sample sizes may necessitate crude groupings of different minority communities into higher level ethnic or even a combined “BAME” (Black, Asian and minority ethnic) group to enable statistical comparison. There is ongoing discussion around the appropriate terminology to use when talking about ethnicity (3, 4). A briefing by the King's Fund (4) highlights the differences between and within communities. For instance, the higher-level group “Asian” tends to include people from such diverse communities as Chinese, Bangladeshi or Indian, the latter in itself including diverse subgroups such as Hindus, Muslims, Sikhs and Christians (4).

Health inequalities have been a significant and longstanding societal challenge in the UK. While people from minority ethnic communities have long been found to experience poorer physical and mental health outcomes (5–7), these inequalities were brought to the fore during the COVID pandemic where people from minority ethnic, including black and Asian, communities were not only more likely to contract but also to die from COVID than those from white communities (8). Similarly, there is evidence that people from minority ethnic communities may be disproportionally affected by visual impairment (V.I.), although the UK does not monitor V.I. in the general and subgroups within the population. As such, prevalence and risk are calculated for smaller samples. Prevalence calculated from estimates published in 1994 and 2004 (9–16) suggest that, while V.I. is projected to increase in all ethnic groups by 2050, minority ethnic communities are projected to make up an increasing proportion of adults living with V.I. (17). More recently, there is evidence from a sample of 503,325 adults aged 40–69 drawn from the UK Biobank, that people from non-white communities are 1.7 times more likely to have mild V.I. (Snellen visual acuity of 6/7.5–6/18) and 1.4 times more likely to have low vision (6/18–6/120) than those from white communities (18). The highest risk groups were “black other” and Bangladeshi communities who were 3.4 and 2.3 times more likely and black African and Pakistani communities who were twice as likely as white British communities to have any V.I. Finally, in a sample of 112,314 participants aged 40–73 also drawn from the UK Biobank (19), the risks of socially significant (<6/12–6/18) and bilateral V.I. (<6/19) were approximately double for all minority ethnic communities relative to white communities, but the risk of bilateral V.I. was slightly lower among Chinese communities and the risk of socially significant V.I. may be lower among mixed ethnic than white communities. The evidence reviewed here highlights the difficulties in comparing results across studies due to the use of different statistics, categories of V.I., and ethnic subgroupings.

V.I. can impact on a wide range of life domains including activities of daily living, such as shopping, self-care and household chores (20, 21), participation in sports and leisure activities (22, 23), and employment outcomes, whereby working-age adults with V.I. may be at increased risk of being unemployed or having a lower-status job (19). Moreover, V.I. has been associated with poorer quality of life, social functioning, sleep quality, mental health outcomes (19, 24–30), and loneliness, particularly in older adults (31–34).

Despite the increased risk of V.I., a review published in 2005 reported that uptake of services for V.I. may be low among people from minority ethnic communities (35). The review suggested this may be due to a lack of cultural sensitivity and relevance, including information materials not being available in different languages, and a lack of cultural competence among professionals. There is tentative evidence that cultural competence training may have a positive impact on patient outcomes (36, 37), but the content of training is variable and may consist of on one or more aspects such as cultural sensitivity, cultural knowledge and awareness, cultural skills such as intercultural communication, and attitudes such as the desire to learn (36–38). In the UK, people living with moderate or severe V.I. can be registered as sight impaired (partially sighted) or severely sight impaired (blind) with their local social services. However, Pardhan and Mahomed (39) found evidence of an underrepresentation of Asian communities registering as sight impaired or severely sight impaired, particularly older Asian adults relative to white communities, and of Asian women relative to Asian men. This is significant because registration provides a number of benefits, including a needs assessment and appropriate support to remain independent, as well as financial concessions on transport, television and health services.

Considering the increasing ethnic diversity in the UK population, the increased risk of V.I. with its wide-ranging impacts, and the possible underutilisation of support services among minority ethnic communities, it is important to have an understanding of the impact of V.I., support needs and the extent to which these are met among different ethnic communities. This research builds on previous work (35) published in 2005 to provide an overview of recent evidence. A rapid evidence review was carried out to explore the current knowledge relating to the wider impact of living with V.I. and experiences along the eyecare pathway of people from minority ethnic communities in the UK.

## 2. Methods

A rapid evidence review has been defined as “*a form of knowledge synthesis in which components of the systematic review process are simplified or omitted to produce information in a timely manner*” [(40), p. 1]. As such it involves a more rigorous and structured process than a standard literature review. There is no agreed process for a rapid evidence review and the exact methodology has been found to vary between rapid evidence reviews (40). Rapid rather than systematic review methods were selected for this project to provide an overview of available evidence in a timely manner to support the work of BAME Vision, which has been set up to address inequalities in eye health care, to raise awareness of support and services, and to tackle misconceptions and break down barriers faced by minority ethnic communities.

The current review involved a systematic search of academic literature in the academic databases AMED, CINAHL Plus and MEDLINE *via* EBSCOhost using the search string:

(Low vision OR vision loss OR reduced vision OR subnormal vision OR diminished vision OR vision impair^*^ OR visual^*^ impair^*^ OR sight loss OR sight impair^*^ OR blind^*^ OR partial^*^ sight^*^ OR purblind OR unsighted OR SSI) AND (BAME OR BME OR ethnic minority OR minority ethnic OR Black OR Asian OR African OR Caribbean) AND (UK OR United Kingdom OR Great Britain OR Britain OR England OR Scotland OR Wales OR Northern Ireland).

**Figure 2:**
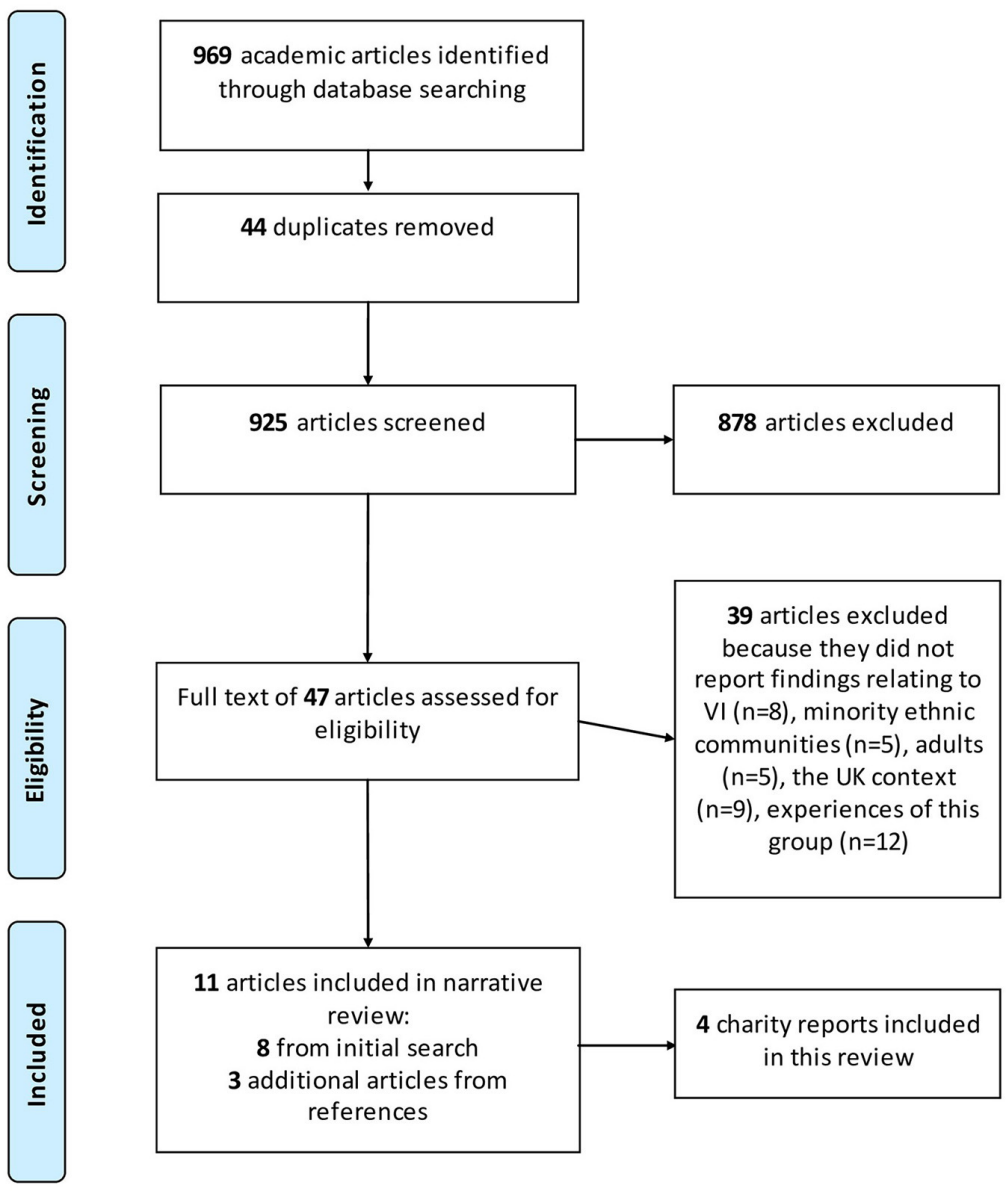
PRISMA flow diagram.

The search was restricted to articles published since 2005, in English, and reporting on research with human participants. The search identified 969 articles which were imported into Covidence systematic review software (Veritas Health Innovation, Melbourne, Australia. Available at www.covidence.org). Relevant articles were identified in two stages. First, the abstract and title of articles were screened against a list of inclusion criteria, then the full text of included articles was reviewed. Two researchers contributed to the initial title and abstract screening and the full-text review. To expedite the screening process, each article was reviewed by one researcher only. After removing 44 duplicates, the abstract and title of 925 articles were screened. Articles were included in the subsequent full-text review if they presented findings relating to the wider impact of V.I. on people from minority ethnic communities in the UK and/or their experiences along the eyecare pathway. Thus, articles which explored optometry service use but contained participants without V.I. were included in the review, due to their relevance for experiences relating to the first stage on the eyecare pathway. No limitations were set on study design so that qualitative, quantitative and mixed-methods research as well as reviews were included. Articles were excluded if they reported findings from outside the UK, did not relate to adults, experiences along the eyecare pathway or of living with V.I., and/or did not present findings for people from minority ethnic communities. Articles using mixed samples consisting of people from white and minority ethnic communities were excluded, if findings for participants from minority ethnic communities were not reported separately from findings for participants from white communities. Where it was unclear if an article was relevant from the title and abstract, the article was included in the full-text review. After excluding 878 irrelevant articles, a total of 47 articles were included in the full-text review. At this stage the full article was read and assessed against the inclusion criteria listed above and data were extracted from relevant articles. A total of 39 additional articles were excluded because they did not report findings relating to V.I. (*n* = 8), people from minority ethnic communities (*n* = 5), adults (*n* = 5), the UK context (*n* = 9) and the experiences of people from minority ethnic communities with V.I. (*n* = 12) (Figure 2). The latter 12 articles predominantly reported findings on genetic variants associated with conditions causing V.I. and prevalence or risk of V.I. and certain eye conditions, but did not provide useful information on experiences of living with V.I. or experiences along the eyecare pathway All excluded articles were reviewed for data which may provide context. Three additional articles were identified in the reference list of the 8 included articles during data extraction.

In addition, a search of the gray literature was conducted. The websites of the Royal National Institute of Blind People (RNIB), the Thomas Pocklington Trust (TPT), Moorfields Eye Hospital and the Office for National Statistics (ONS) were searched for reports presenting findings relating to the experiences of people from minority ethnic communities living with V.I. in the UK. The RNIB, TPT and Moorfields Eye Hospital were also contacted and asked for any additional reports that were not available on their website. The findings from one report on the RNIB website (41) was also presented in an academic article (42). Findings are reported from the peer-reviewed academic journal article.

Data was extracted from a total of 11 academic articles and four charity reports and presented as a narrative review. Articles were not quality assessed but limitations were noted. The findings were written up in a detailed (unpublished) report for BAME Vision and are summarized in the current manuscript. The articles were grouped into broader topic areas to organize findings thematically.

## 3. Results

Five themes were identified in the 11 academic articles and four reports from the gray literature included in this review (some articles are included in multiple sections): Perceptions of V.I. and eye disease (*n* = 3), Access to services and service use (*n* = 5), Impact of interventions (*n* = 7), and Wider impact of V.I. (*n* = 2). One article explored registration status. The following sections provide an overview of available evidence relating to experiences of services, support and needs along the eyecare pathway and highlight gaps in the literature.

### 3.1. The eyecare pathway

In the UK, primary care services such as community optometry and general practice are typically the first point of contact on the eyecare pathway for people with and without vision or eye problems. Amendments to statutory legislation have enabled some patients to be referred to specialist optometrists in the community for disease management and access to some therapies. As a result, the scope of the role for primary eyecare services has been extended considerably (43, 44). Individuals with eye problems beyond the scope of community optometry will usually be referred to hospital-based ophthalmology services, where a range of medical and surgical interventions can be explored, and patients are signposted to support services.

### 3.2. Optometry services

Much of the research on service use since 2005 has focused on identifying barriers and facilitators to uptake of eye tests among different groups, including Afro-Caribbean, Pakistani, Somali and Gujarati communities. Common barriers identified across the research include cost and the retail context of community optometry [“*… He just gets on with what he needs to do. We talk about my glasses really. He seems to be more interested in selling me new ones*”, Cross et al. (45), p. 917], dissatisfaction with prior primary care experiences, cultural barriers, including language, particularly among older adults, and negative perceptions of V.I. and eye conditions (45–47). Although the latter may not only delay service use but also impact on how well people adapt to sight loss, there was limited research on how different communities perceive V.I. Patel and colleagues (48) found that members of the Indian community in Ealing, West London, viewed sight loss as being part of the aging process, becoming a major disability only once there was substantial dependence and loss of function. Members of Somali, African-Caribbean and Gujarati communities described sight loss as devastating and resulting in dependence on others (47), and it was associated with being a victim, helplessness and social isolation by members of the Afro-Caribbean communities (49). The latter gives some insight into how people living with V.I. are perceived within Afro-Caribbean communities. However, similar to perceptions of V.I., perceptions of people living with V.I. among different communities remain underexplored, despite the potential impact negative attitudes may have on areas such as participation in everyday life (50) and identity (51). Limited awareness of eye health was found to be a substantial barrier and may have resulted in eye health not being considered a priority or forming part of a healthy lifestyle. For instance, prevalence of primary open-angle glaucoma has been found to be highest among adults from Afro-Caribbean communities in all age groups (16, 52). However, while awareness of glaucoma was high among a sample of adults from Afro-Caribbean communities, descriptions of glaucoma tended to be vague [“*weakness in the eye*” or “*something to do with skin over the eyes*”, Cross et al. (49), p. 84]. More detailed descriptions (“*build-up of pressure due to lack of drainage*”, p. 84) were found among the small proportion who had heard about glaucoma from their optometrist (49). Importantly, glaucoma was associated with aging resulting in low perceived risk of experiencing glaucoma at a younger age (49).

A number of pilot studies have also assessed the efficacy and cost-effectiveness of interventions such as offering free glaucoma tests in GP practices (42) and using community eye champions (47) in increasing uptake of eye examinations. However, there was no evidence available relating to the outcomes of longer-term interventions. Taken together the research carried out to date provides a comprehensive list of barriers to service use and suggestions to overcome these (Table 1). These include fostering trust and positive clinician-patient experiences to overcome negative prior experiences, and providing information about services which are free to overcome barriers relating to cost and the retail context of optometry services. Suggestions to raise awareness of eye health included teaching people about eye health from an early age by putting it on the school curriculum and drawing on trust relationships with GPs and community partners to disseminate eye health information. Suggestions to increase the cultural appropriateness of services included hiring staff from different ethnic communities and providing information in different languages (45–47). Clinicians and public health officials may also need to take into account age (e.g., younger people tended to prefer quicker, drop-in, express examinations, whilst those aged 30+ tended to prefer more thorough eye examination with greater information being provided) (45) and ethnic group differences (e.g., the Somali community tends to share information verbally and may prefer eye health information being shared in group interventions) (47) when designing eye health information and services. Finally, reminder letters or calls may be effective in increasing attendance (42, 46). There was no evidence relating to the experiences of receiving further eyecare by specialist optometrists in the community.

**Table 1:** Common barriers and recommendations to increase uptake of primary eyecare services.

**Barriers**	**Recommendations**
Limited awareness of eye health	• Put eye health on the school curriculum • Make information available in different languages, culturally appropriate and accessible • Draw on trusted relationships (GPs) and community partners to disseminate eye health information
Cost and retail context of optometry	• Include information about free services in eye health messaging • Train optometry staff to provide information about free services
Dissatisfaction with previous experiences	• Build trust by spending time with patients • Improve communication from clinical/non-clinical staff
Cultural barriers	• Provide information in different languages • Recruit staff from ethnic communities • Work with community partners

### 3.3. Hospital and specialist optometry eyecare services

Less research has focused on experiences following referral into secondary or tertiary eyecare services. Evidence from a pilot intervention testing the efficacy of offering free glaucoma checks in GP practices to black communities found that six of the 28 patients (21.4%) who were referred on to Moorfields Eye Hospital did not attend a hospital appointment to be assessed for glaucoma (42). Only one article explored barriers to use of further eyecare services and only among Indian communities (48). These barriers largely reflect issues identified for optometry service use. Limited awareness of potentially sight-threatening eye conditions may result in a symptom-led use of services: there was a tendency to delay treatment until conditions were no longer manageable. The authors gave the example of cataracts, which were thought to require an undetermined period of maturing or “*ripening*” until the condition was no longer manageable, before individuals sought treatment. This is of note because it may result in people seeking treatment for avoidable sight loss once vision has substantially deteriorated, which in some conditions is non-recoverable. There was also dissatisfaction with prior health service experiences, including the quality of care received and long waiting times, and limited awareness of how to access services, with GPs being seen as gatekeepers. Along with limited acceptance and trust of Western medicine, this resulted in some seeking treatment abroad where they could choose when and with whom to have treatment. Additional barriers were: lack of time, health not being seen as a priority among a population of immigrants who came to the UK for socio-economic reasons, and fear of the unknown arising from a poor understanding of eye conditions and possibly compounded by unhelpful perceptions of sight loss and long waiting times for appointments and treatment. Language may present an issue once patients had accessed services and were reliant on interpreters for communication with medical professionals (48). The article did not explore facilitators to service use. There was no evidence relating to the experiences of other ethnic communities, of receiving hospital eyecare as an outpatient, and of receiving further care by specialist optometrists in the community.

### 3.4. Treatment

Some eye conditions can be treated to delay or avoid sight loss. Despite increasing availability of therapeutic and surgical interventions for chronic eye conditions such as glaucoma and neovascular age-related macular degeneration, there is anecdotal evidence from the gray literature that older people from minority ethnic communities may be less aware of treatment options (53). In addition, there is some evidence that certain common ophthalmic treatments may not be universally effective. For example, vitrectomy (54, 55) and injections (56) for diabetic eye disease were found to be effective among patients from Asian communities, but vitrectomy may be less effective in diabetics from Afro-Caribbean communities. Gupta et al. (54) observed smaller increases in visual acuity following vitrectomy for tractional retinal detachment and other complications associated with end-stage diabetic retinopathy (but not non-clearing vitreous hemorrhage) among this group, who were also more likely to require further surgery for complications such as a re-detached retina, non-clearing vitreous hemorrhage, or epi-retinal membrane (24% compared to 21% of patients from white and 14% from South Asian communities). Moreover, Mastropasqua et al. (55) also reported significantly poorer surgical outcomes following vitrectomy for traction complications related to proliferative diabetic retinopathy among patients from black communities: 43% required a silicone oil tamponade during surgery (compared to 17% of patients from South Asian and 16% of patients from white communities) and 30% required long-term silicone oil tamponades of more than 6 months (compared to 9% of patients from South Asian and 7% of patients from white communities). But unlike Gupta et al. (54), they found a decrease in visual acuity in this group. Although it must be acknowledged that this does not constitute a comprehensive review of the efficacy of various treatments in different ethnic groups, these findings do provide some indication that ophthalmic consultants may need to consider ethnicity and possible differences in side effects and efficacy when considering treatment options and discuss these with patients.

### 3.5. Wider support

When people start to lose their sight, they may need support to help them adapt to sight loss. As seen earlier, V.I. can impact on a wide range of areas including activities of daily living (20, 21), participation in sports and leisure activities (22, 23), quality of life, sleep, mental health and social functioning (19, 24–30). However, there has been relatively little research since 2005 exploring the wider impact of V.I. on the everyday lives of and use of wider support services among people from minority ethnic communities. One review article identified unmet needs relating to isolation and self-esteem among people from minority ethnic communities (57). Although working-age adults with V.I. have been found to be at increased risk of being unemployed and having a lower-status job (19), and despite the potential for race and disability discrimination, a charity report found no statistically significant association between employment and ethnicity in a survey of more than 1,200 people registered as blind or partially sighted in the UK, 703 of whom were of working age (58). However, the report provided no information about other employment outcomes. Qualitative research found that older adults with V.I. regardless of ethnicity share difficulties relating to activities of daily living, mobility outside the home, maintaining control and independence, and a diminishing social network, but older adults from minority ethnic communities were also found to be less likely to have up-to-date technological devices and to leave the home; and they were more likely to have help from family members with everyday tasks than those from white communities (53). No research on the impacts on quality of life, mental health, sports and leisure activities, sleep quality, identity and social and romantic relationships were identified in the search.

Early intervention services such as the Eye Clinic Liaison Officer (ECLO) can provide important emotional and practical support following a diagnosis of irreversible sight loss, and bridge the gap between health and wider support services (59). Being based in eye clinics, ECLOs provide advice and support relating to a wide range of needs including registration of a V.I., hospital appointments, welfare benefits, education, employment, housing, low vision aids or training, travel and social networks and by referring and signposting patients and their families to social services, sight loss charities or support groups (60). However, not all eye clinics have early intervention support such as an ECLO and minority ethnic communities may be underrepresented in these services, with 96.4% of service users being from white communities and only 3.6% from black and other minority ethnic communities (59).

Despite the benefits of registering a V.I. in the UK, only one article assessed the prevalence and risk factors associated with under-registration. Reviewing the medical records of 2,161 adult patients attending ophthalmology outpatient services, Barry and Murray (61) found that 18 of the 56 (32.1%) patients who were eligible to be registered as severely sight impaired (blind) and 47 of the 90 (52.2%) patients who were eligible to be registered as sight impaired (partially sighted) were unregistered. People from minority ethnic communities were around three times more likely to be unregistered than those from white communities (OR=3.23, 95% CI: 1.56–6.65, *p* = 0.0015). Other risk factors included being sight impaired (vs being severely sight impaired), having had 4 or fewer appointments at the hospital (vs having had 5 or more appointments) and having a treatable condition (vs. having an untreatable condition). Overall, this suggests that those with milder levels of V.I. may be more likely to be unregistered. In addition, 13 of the 38 patients who were registered as severely sight impaired and 19 of the 38 patients who were registered as sight impaired were registered inappropriately based on their recorded level of vision. Being aged 65 and over was associated with being registered incorrectly. The article does not explore why some patients were registered incorrectly, such as clerical errors, or changes in the degree of V.I. since initial registration. There was no association between the grade of the examining doctor and registration status. Awareness of the registration process was low among doctors of all grades. The authors suggest that ethnic group differences in registration may be due to inhibitions, particularly among older adults, and/or communication difficulties between clinicians and patients resulting in lower levels of awareness and knowledge of the benefits and processes involved in registering a V.I. This suggests that work is required to increase awareness of the register and the benefits associated with registration among clinicians and patients alike.

Sight loss charities can provide practical support and guidance including vision rehabilitation and mental health support going forward. However, there is anecdotal evidence that older adults from minority ethnic communities may be less aware of wider support services and how to access these, and they may be more likely to attend V.I. groups specifically for their ethnic community despite efforts by national sight loss charities to reach them (53). Only one review article provided a list of unmet needs, including a lack of knowledge and understanding of eye conditions, services and benefits, registration and the benefits of rehabilitation, and a set of recommendations for wider support services including sight loss support and housing providers (57). Recommendations consisted of hiring staff from minority ethnic communities, working actively with families in need, providing materials in different languages, working with community partners to disseminate information, providing adequate funding and resources to partners offering support services within their communities, and working to build relationships and overcome negative experiences by providing a continuous service (57). Guidance for eyecare and sight loss support providers on how to increase the cultural sensitivity and appropriateness of their services are also available from organizations such as BAME Vision.

One area of support provided by sight loss charities is vision rehabilitation through training, equipment or mobility aids such as guide dogs or canes to help people maintain their mobility and independence. However, there is some evidence that use of mobility aids, training and equipment may be lower among certain communities (45, 47, 57). For instance, a guide dog may not be appropriate for members of the Somali community [“*It would be especially difficult in our community, as they won't want to have guide dogs, like other communities and so you will not be able to go out and do things. You will be stuck at home*.”, Biddyr et al. (47), p. 38] and members of the Afro-Caribbean communities (45). This is an important area of research, which will need to identify the acceptability of available training, equipment and aids within different communities and alternatives that will enable people to remain independent and mobile.

## 4. Discussion

This review provides an overview of research published in the last 17 years relating to the wider impact of V.I. and the experiences of people from minority ethnic communities along the eyecare pathway in the UK. While several studies have explored barriers and interventions to increase primary eyecare service use, particularly eye examinations to detect certain eye conditions and V.I., fewer have explored experiences of tertiary eyecare and wider support. This focus on primary eyecare may be partly driven by necessity, with detection being the first step on the eyecare pathway. The research has produced a comprehensive list of barriers and suggestions on how barriers may be mitigated. While small-scale interventions have tried to increase service use by increasing awareness of eye health and bringing eye examinations into a familiar clinical context (e.g., GP practice), there are other barriers, such as unhelpful perceptions of V.I. which have received less attention. The results indicate that there are a number of implications for practitioners working with people from minority ethnic communities with V.I. First, recommendations for service providers along the eyecare pathway concur about the importance of taking time with patients, clear communication and building trust to overcome previous negative experiences as well as raising awareness of eye health, increasing cultural representation among clinical and non-clinical staff, and working with community partners to support communities. Second, two articles in this review identified ethnic group differences in the efficacy of a common treatment for diabetic eye disease. Although this does not constitute a comprehensive review of the efficacy of treatments, it does indicate that ophthalmic consultants may need to consider ethnicity when recommending treatment options for certain eye conditions. Third, cultural differences in the acceptability of guide dogs suggest that support organizations may need to work with communities to identify appropriate mobility aids and, where required, suitable alternatives to ensure people retain their mobility and independence. Fourth, a review of UK-based primary care and hospital databases showed that usable ethnicity data was available for only 46.8% of outpatients in 2011, even where systems were in place to record such information (62). Without complete ethnicity data, it is not possible to effectively evaluate service use, health outcomes, and the extent to which services are universally appropriate. Therefore, greater attention is needed to maintain accurate and complete service user records, where demographic information such as ethnicity is systematically captured. This applies to healthcare as well as wider sight loss support services.

There are also clear implications for researchers. Overall, the findings suggest that much of the evidence remains anecdotal, outdated or unavailable. For example, 6 of the 15 articles included in this review (40%) were published 10 years ago (2012 or earlier). The most recent articles were published in 2017 and explored employment status, the efficacy of vitrectomy, and the efficacy of offering glaucoma checks in GP practices. There are substantial gaps in the evidence relating to perceptions of V.I. and people with V.I. in different communities, and how these impact on treatment-seeking and adaptation to sight loss. This review also found gaps in the literature relating to the wider impact of V.I. The scarcity of research relating to the wider impact of V.I. on the lives of people from minority ethnic communities in the UK and the extent to which their support needs are met should not, in the words of Johnson and Morjaria-Keval (57), be equated to “*evidence of an absence of need*” (p. 22). There is evidence that adults with V.I. from black and Asian communities may be younger and may therefore have different support needs to those from white communities. For instance, prevalence of primary open-angle glaucoma is highest among black communities in all age groups but there may be a greater increase in prevalence with increasing age among white communities (52). Moreover, Asian ethnicity has been associated with an increased risk and earlier onset of diabetic retinopathy (39), sight-threatening diabetic retinopathy (63) and cataract (9) compared to white ethnicity. This suggests that people from minority ethnic communities may experience V.I. at different life stages to those from the majority white communities, resulting in different support needs. There is therefore a clear need to further explore the experiences and support needs of different ethnic communities and the extent to which these needs are met by service providers.

Further work is needed to explore barriers and facilitators to use of eyecare services other than community optometry. Anecdotal evidence suggests that not all available mobility aids may be universally acceptable; thus, research is required to explore the acceptability of existing mobility aids within different communities and the potential alternatives to minimize the impact of V.I. Finally, considering the heterogeneity within and between ethnic communities (4), research needs to focus on the experiences of individual ethnic communities along the eyecare pathway, similar to the work of the ReGAE (research into glaucoma and ethnicity) project which explores experiences of glaucoma among Afro-Caribbean communities in the UK [e.g., (45)].

### 4.1. Limitations

The research reviewed here includes academic articles and reports published since 2005, in English and for UK findings only. Not all organizations contacted for gray literature responded. Contrary to systematic reviews, the reference lists of included articles were not reviewed systematically for potentially relevant articles to facilitate a timely delivery of findings, although a number of additional articles were identified and included during the full text review. This article aims to provide practical implications for practitioners and researchers. However, the limited evidence available, the anecdotal nature of some findings, and the time since research has been completed must be kept in mind when reviewing these recommendations.

Even though the research suggests that there are important group differences, it was not within the scope of this article to provide a profile of the experiences of individual ethnic communities. This partly reflects the tendency in the research literature to compare white to non-white/minority ethnic communities to account for small subsample sizes. Neither was it possible to compare experiences for those with acquired vs. congenital sight loss, by severity of sight loss or for different eye conditions. These will be important areas of research for the future.

### 4.2. Conclusions

Considering the increasing ethnic diversity in the UK and the increased risk of V.I. among minority ethnic communities, there is an urgent need to fill the gaps in the research identified here to gain a better understanding of the experiences of what is an increasing number of people living with V.I. to ensure their needs are met.

## Author contributions

NH conducted the literature search and data extraction. LJ and BM reviewed and commented on the data. NH and LJ drafted the initial article. All authors reviewed and edited the article, approved the final version to be published, and agreed to be accountable for all aspects of the work.
